# Supplementary figures and images for: Airway epithelial specific deletion of Jun-N-terminal kinase 1 attenuates pulmonary fibrosis in two independent mouse models
Source: PLoS One. 2020 Jan 14;15(1):e0226904. doi: 10.1371/journal.pone.0226904 (PMC6959564; doi:10.1371/journal.pone.0226904)

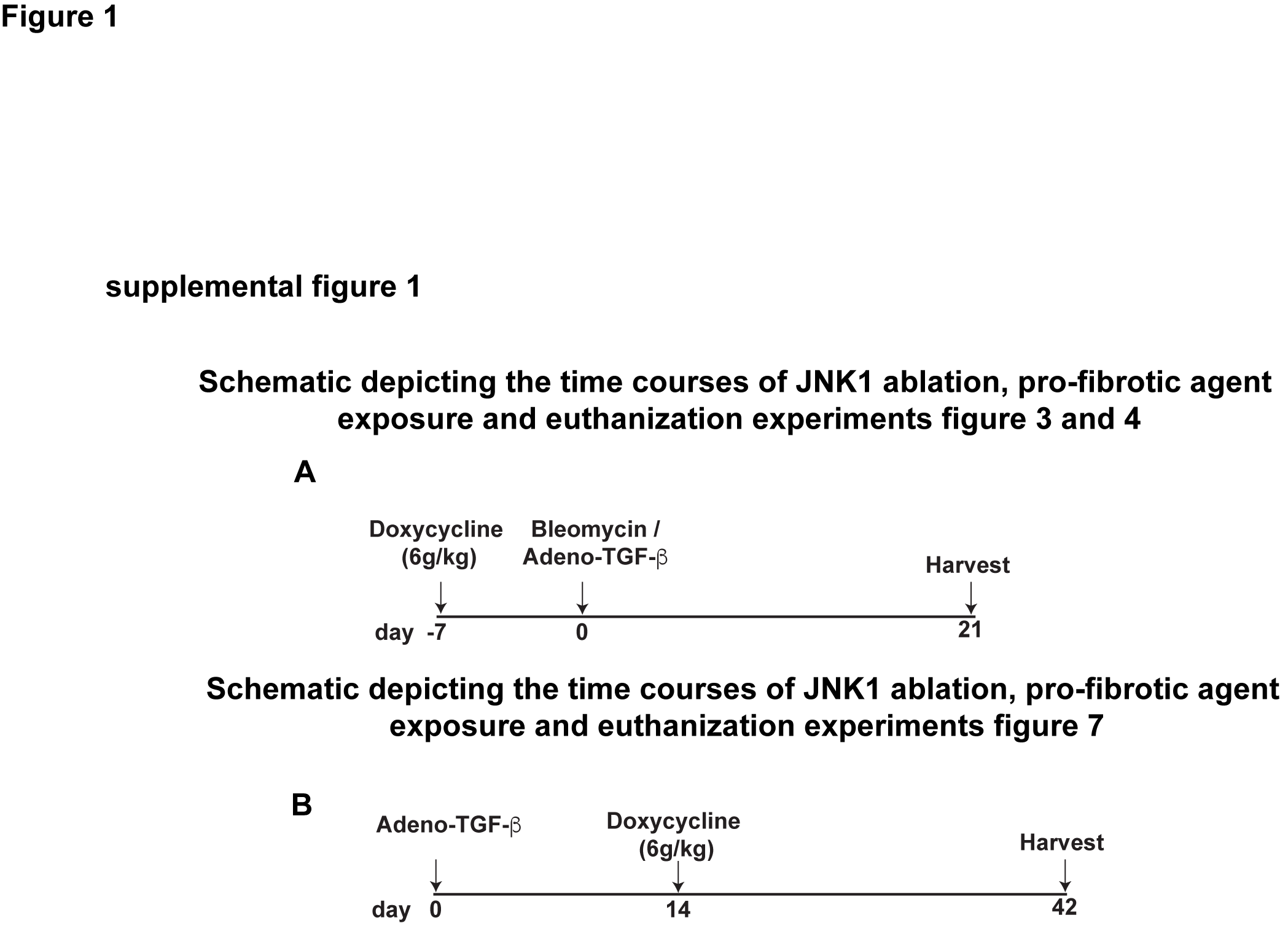

Supplement: S1 Fig — (TIF) [file pone.0226904.s001.tif]
