# Supplementary material for: Airway epithelial specific deletion of Jun-N-terminal kinase 1 attenuates pulmonary fibrosis in two independent mouse models
Source: PLoS One. 2020 Jan 14;15(1):e0226904. doi: 10.1371/journal.pone.0226904 (PMC6959564; doi:10.1371/journal.pone.0226904)

UNEDITED GEL FOR FIGURE 1

P-JNK1/2

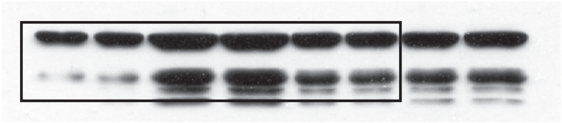

JNK1/2

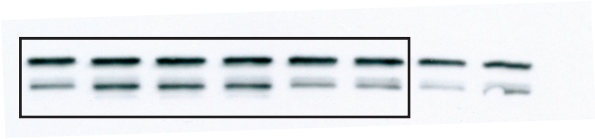

JNK1/2

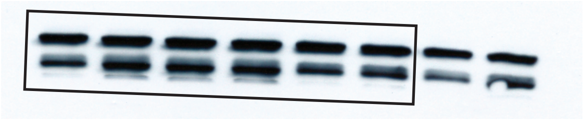

longer exposure

UNEDITED GELS FOR FIGURE 2

JNK1/2

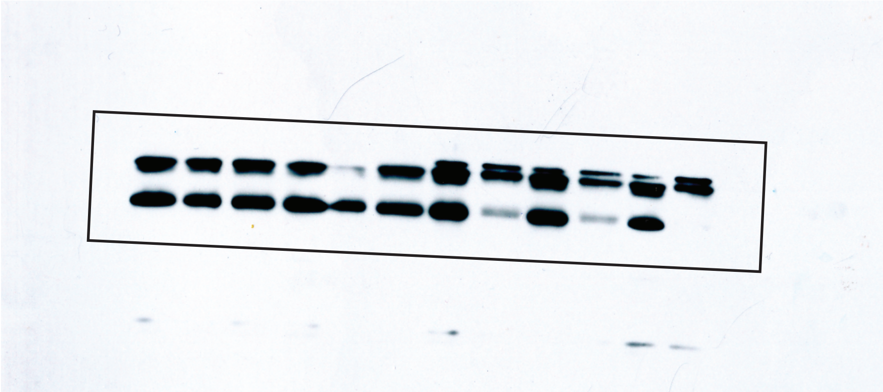

actin

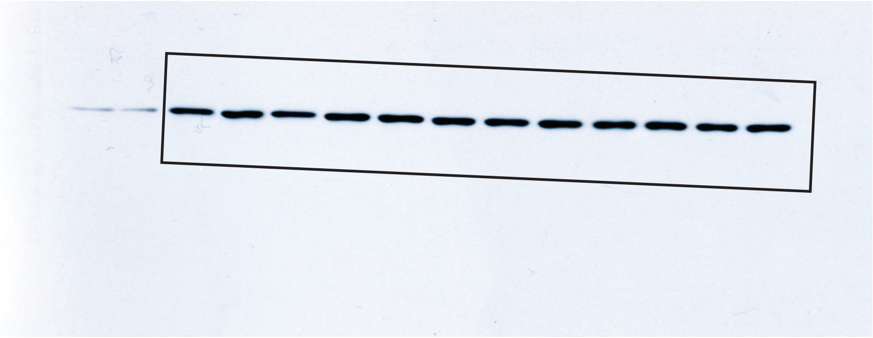

UNEDITED GEL FOR FIGURE 5

α-SMA 42kd

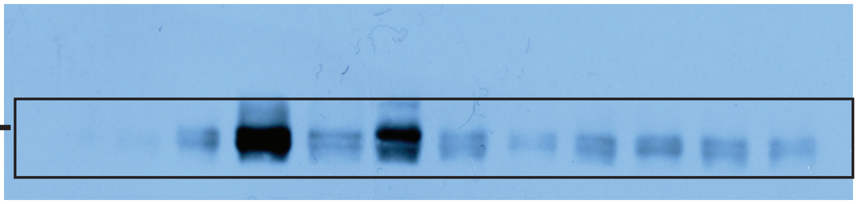

Col1a1 140kd

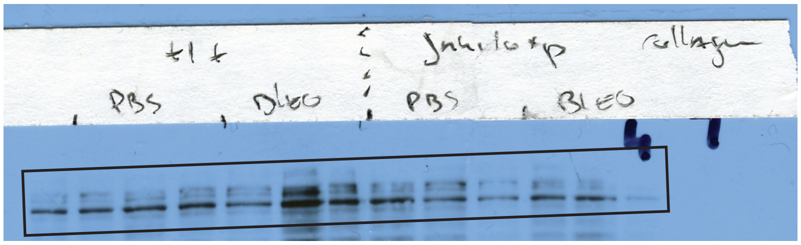

FSP1 (S100a4) 12kd

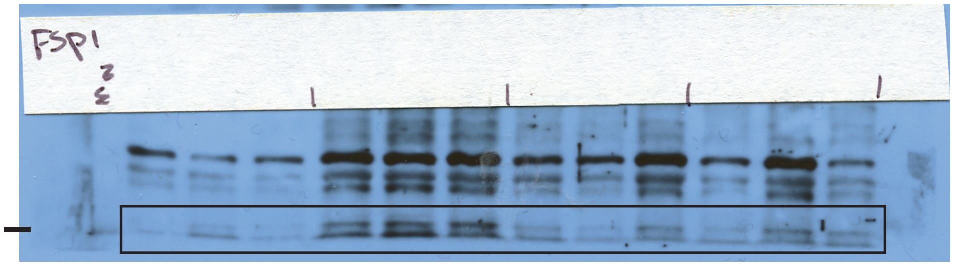

actin 42kd

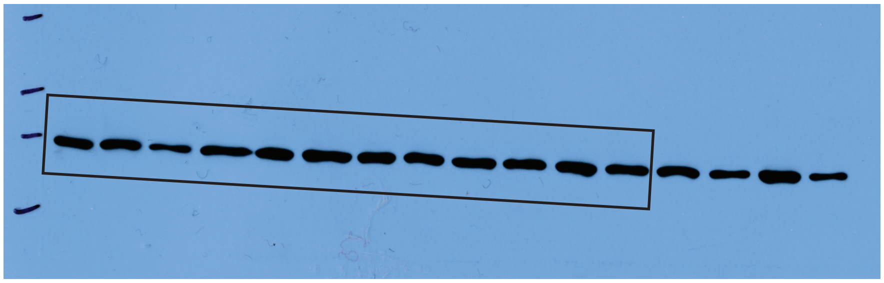

UNEDITED GELS FOR FIGURE 6

α-SMA 42kd

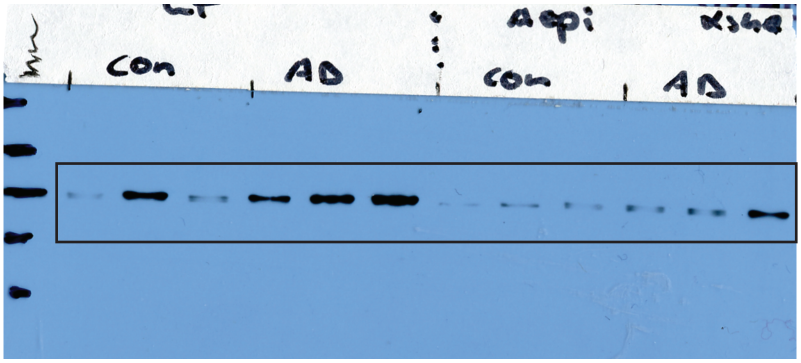

Col1a1 140kd

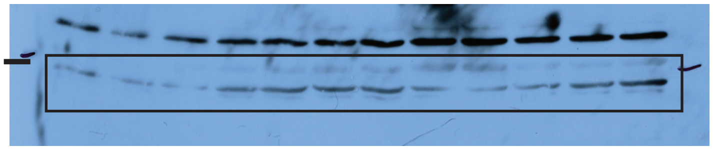

actin 42kd

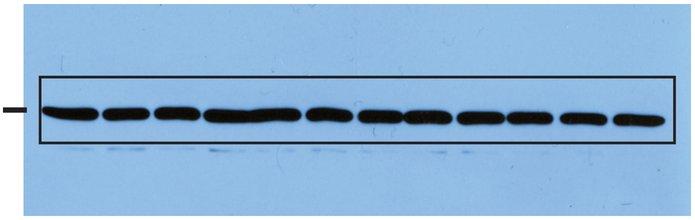

Supplement: S2 Fig — (PDF) [file pone.0226904.s002.pdf]
